# Combination of thermal ablation by focused ultrasound, pFAR4-IL-12 transfection and lipidic adjuvant provide a distal immune response

**DOI:** 10.37349/etat.2022.00090

**Published:** 2022-06-29

**Authors:** Hai Doan Do, Corinne Marie, Stéphanie Bessoles, Hélène Dhotel, Johanne Seguin, Benoit Larrat, Bich-Thuy Doan, Daniel Scherman, Virginie Escriou, Salima Hacein-Bey-Abina, Nathalie Mignet

**Affiliations:** 1 Université de Paris Cité, CNRS, INSERM, UTCBS, 75006 Paris, France; 2 Chimie ParisTech, Université PSL, F-75005 Paris, France; 3 NeuroSpin, Institut des Sciences du Vivant Frédéric Joliot, Commissariat à l’Energie Atomique et aux Énergies Alternatives (CEA), Université Paris Saclay, 91191 Gif-sur-Yvette, France; 4 Université PSL, Chimie ParisTech, CNRS, SEISADCNRS, 75005 Paris, France; 5 Clinical Immunology Laboratory, Groupe Hospitalier Universitaire Paris-Sud, Hôpital Kremlin-Bicêtre, Assistance Publique- Hôpitaux de Paris, 94275 Le-Kremlin-Bicêtre, France; Donghua University, China

**Keywords:** Gene delivery, cancer, transfection, immunotherapy, Toll-like receptor 2-agonists, combined therapy, therapeutic ultrasound

## Abstract

**Aim:**

Gene-based immunotherapy against cancer is limited by low gene transfer efficiency. In the literature, interleukin-12 (IL-12) encoding plasmid associated with sonoporation has been shown to enhance antitumoral activity. Moreover, non-viral carriers and high-frequency ultrasound have both been shown to promote immune response activation. Here, IL-12 encoding plasmid, non-viral carrier stimulating the immune response and focused ultrasound were combined in order to improve the antitumoral efficiency.

**Methods:**

In order to enhance a gene-based antitumoral immune response, home-made lipids Toll-like receptor 2 (TLR2) agonists and plasmid free of antibiotic resistance version 4 (pFAR4), a mini-plasmid, encoding the IL-12 cytokine were combined with high-intensity focused ultrasound (HIFU). The lipid composition and the combination conditions were selected following *in vitro* and *in vivo* preliminary studies. The expression of IL-12 from our plasmid construct was measured *in vitro* and *in vivo*. The combination strategy was evaluated in mice bearing colon carcinoma cells (CT26) tumors following their weight, tumor volume, interferon-gamma (IFN-γ), and tumor necrosis factor-alpha (TNF-α) levels in the serum and produced by splenocytes exposed to CT26 tumor cells.

**Results:**

Lipid-mediated cell transfection and intratumoral injection into CT26 tumor mice using pFAR4-IL-12 led to the secretion of the IL-12 cytokine into cell supernatant and mice sera, respectively. Conditions of thermal deposition using HIFU were optimized. The plasmid encoding pFAR4-IL-12 or TLR2 agonist alone had no impact on tumor growth compared with control mice, whereas the complete treatment consisting of pFAR4-IL-12, TLR2 lipid agonist, and HIFU limited tumor growth. Moreover, only the complete treatment increased significantly mice survival and provided an abscopal effect on a metastatic CT26 model.

**Conclusions:**

The HIFU condition was highly efficient to stop tumor growth. The combined therapy was the most efficient in terms of IL-12 and IFN-γ production and mice survival. The study showed the feasibility and the limits of this combined therapy which has the potential to be improved.

## Introduction

Using a gene as a drug was first proposed in the 1960s. DNA has been initially proposed as a medicine to treat the genetic disorder in rare diseases often lacking enzymatic expression [1]. Since the first success, many nucleic acid-based treatments have been taken into clinical trials in particular thanks to the improvement of technologies to produce efficient and safe vectors [2]. Since then, the interest of nucleic acids as medicines has been enlarged to other diseases, in particular cancer. Until 2016, approximately 1,600 of the 2,600 gene therapy clinical trials were performed in the field of oncology [3]. In the immunotherapy field, gene therapy plays a critical role, with examples of *in vivo* delivery of cytokine genes to tumors, generating genetically modified tumor or dendritic cells (DCs). Interleukin-12 (IL-12) plays an important role in the induction of both innate and adaptive immune responses and has been produced by antigen presenting cell (APC)-like macrophages and DCs [4]. Indeed, IL-12 stimulates the proliferation and cytotoxic activity of natural killer (NK) cells and induces the production of chemokines that attract different types of immune cells to the tumors. In addition, IL-12 contributes to the maturation of activated T lymphocytes to cytotoxic T lymphocytes (CD8^+^) and T-helper 1 (Th1) cells (CD^+^) which produce other cytokines such as interferon-gamma (IFN-γ), IL-2, and tumor necrosis factor-alpha (TNF-α). In the context of gene therapy, the DCs modified by the *IL-12* gene and pulsed with tumor antigen-induced lymphocyte proliferation, as well as antigen-specific cytotoxic T lymphocytes activation and antitumor effects against human lung cancer cells [5]. In terms of cytokine gene delivery, besides *ex vivo* transduced-autologous or allogenic tumor cells or other cell types, approaches such as local injection of gene delivery vectors into the tumor mass or its periphery have been reported [6]. For instance, electroporation of IL-12 plasmid (pIL-12) in patients with metastatic melanoma showed a complete regression for 2 patients, and disease stabilization for 8 of 19 patients in the phase I clinical trial [7]. Another example is the combination of local administration of pIL-12 with microbubbles and the application of ultrasound, which, thanks to the cavitation of microbubbles resulted in a significant tumor growth inhibition [8]. Moreover, non-viral vectors have shown antitumor activity in rodent tumor models with pIL-12 condensed with cationic lipids or polymers [9]. Physical, viral, and non-viral gene delivery techniques have been reviewed previously, indicating the interest in combination therapies which lower the threshold for IL-12 efficacy and prevent the toxicity mediated by this cytokine [10].

Interestingly, cationic lipids, which are required for DNA complexation, are also able to activate the immune responses via the Toll-like receptor (TLR) pathway inducing the release of cytokines and chemokines such as TNF-α, IL-1β, and IL-6 [11]. The maturation of DCs induced by cationic lipids has been evidenced by CD80/CD86 expression and has been shown to be dependent on the structure of the lipid. Cationic lipids of the ethylphosphocholine (EPC) family, [1,2-dimyristoyl-sn-glycerol-3-ethylphosphocholine (DMEPC), 1,2-dioleoyl-sn-glycerol-3-ethylphosphocholine (DOEPC), etc.] were more efficient activators than those of the trimethylammonium-propane (TAP) family [1,2-dimyristoyl-3-trimethylammonium-propane (DMTAP), 1,2-dioleoyl-3-trimethylammonium-propane (DOTAP), etc.] (Figure S1). Moreover, lipids with short saturated (C12:0 or C14:0) or long unsaturated (C18:1) chains were stronger activators than lipids with long saturated acyl chains (C16:0 or C18:0) [12]. The lipopolyamines that we had designed in previous studies [13], with a carbon chain of C14, were also shown to be able to activate both TLR2 and TLR4 or NOD-like receptor protein 3 (NLRP3) leading to the production of TNF-α, IL-1β, IL-6, and IFN-γ [14]. The immunostimulating mechanism of these lipopolyamines is different from bacterial pathogen-associated molecular patterns (PAMPs) and is probably linked to their fusogenic properties [15]. As more and more evidence of immunomodulation effects of non-viral DNA vectors has been reported, these effects must be taken into accounts when developing lipid nanoparticles (NPs) for gene-based immunostimulation.

Non-viral vectors by themselves are not able to induce a sufficient *in vivo* gene delivery. Low endosomal escape and the nuclear barrier are still considered as major limitations for non-viral vectors. Endosomal escape and triggered release of the entrapped gene at the target site therefore, are important requirements for an effective gene-based treatment. Intracellular triggers still face many limitations due to the heterogeneity of the affected tissue, which is strongly dependent on the patient’s conditions and disease stage. In opposite, exogenously triggered nucleic acid release is time and site-specific since it is controlled through active management of external stimuli. Non-invasive external signals like light, heat, magnetic field, or ultrasound can be applied to the target site from an outside source in a controlled manner [16]. The signal can induce changes in the stability of the delivery system to provoke endosomal escape and DNA release, but according to the parameters chosen, it can also by itself induce modification of the microenvironment and lead to an additional impact. For instance, when ultrasounds are applied to a tissue, hyperthermia, acoustic pressure, and cavitation may be generated leading to various bioeffects. Consequently, ultrasound fosters perfusion, blood vessel permeability, and cellular uptake of drugs, as it leads to mechanical flows and physical forces carrying nanometric drugs through the blood vessel wall [17]. Besides, hyperthermia is one of the most reported additional tools in drug delivery. Apart from improving drug delivery, an additional therapeutic effect has also been reported in terms of activation of the antitumor immune response [18]. In particular, high-intensity focused ultrasound (HIFU) releases tumor-associated antigens and increases the infiltration and activation of immune cells [19]. Moreover, this thermal ablation may trigger a systemic antitumor response and consequently an immunosuppressive environment through regulation of Th17/regulatory T (Treg) cells imbalance [20] or associated with downmodulation of peripheral CD25^+^ forkhead box protein P3^+^ (Foxp3^+^) Treg cells [21]. Several authors recently reviewed another example of the impact of thermal ablation on the immunosuppressive environment [22, 23], nevertheless, these effects are not sufficient by themselves to prevent tumor recurrence.

Based on all these elements, we presently propose a combination of both hyperthermia and cytokine-based gene delivery. Our original idea was to pre-stimulate immune cells by injection of a TLR2 lipid agonist and DNA-encoding the cytokine IL-12, then capitalize on the capacity of HIFU to promote antigen release by immunogenic cell death. We hypothesized that the combination of these three mechanisms could induce a long-term and specific anti-tumor response. What we present here is the set-up of these experiments to evaluate the feasibility and overcome the difficulties encountered by this combined procedure.

## Materials and methods

### Materials

1,2-dioleoyl-sn-glycero-3-phosphoethanolamine (DOPE) and extrusion membranes were purchased from Avanti Polar lipids, Quant-iT™ PicoGreen^®^ from Invitrogen, luciferase (Luc) assay system from Promega, Pierce™ BCA Protein Assay Kit from Thermo Fisher Scientific, Resazurin sodium salt from Sigma-Aldrich, and D-luciferin potassium salt from Interchim Fluoprobes. CT26 murine colorectal carcinoma cell line [CT26. WT: American Type Culture Collection (ATCC)^®^ CRL-2638™] was purchased from ATCC (LGC Standards, Molsheim, France). Dulbecco’s modified Eagle’s medium (DMEM) completed with 10% fetal bovine serum (FBS), *L*-glutamine (29 mg/mL), penicillin (50 U/mL), and streptomycin (50 U/mL; Gibco, ThermoFisher).

### Cationic Lipids

**Figure 1.**
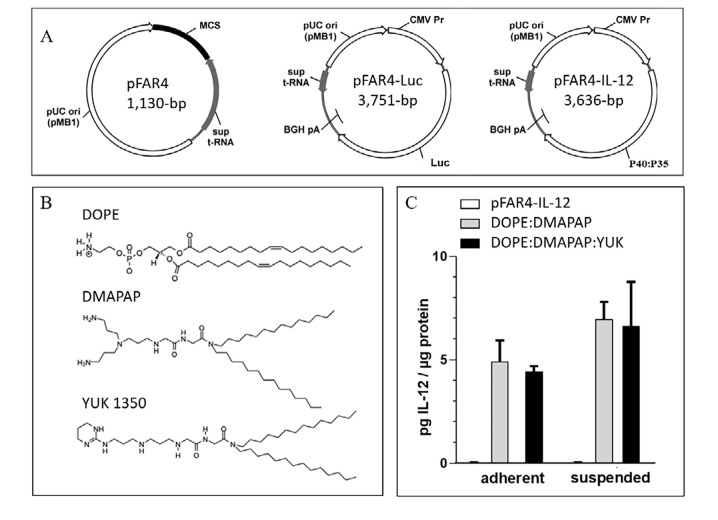
Lipid and plasmid used and their transfection efficiency. (A) Maps and plasmid sizes of pFAR4, pFAR-Luc, and pFAR4-IL-12 containing the Luc and IL-12 expression cassette introduced into the MCS of the pFAR4 gene vector. (B) Chemical structure of the lipids used DOPE, DMAPAP, and YUK1350 in the liposomal composition. (C) Transfection efficacy of adherent or suspended CT26 cells using either naked pFAR4-IL-12, DOPE/DMAPAP, or DOPE/DMAPAP/YUK1350 containing lipoplexes at a dose of 1μg plasmid/well and a charge ratio N/P = 6. After 24 h, transfection efficiency was assessed by quantifying secreted IL-12 levels normalized by total intracellular proteins. The data given are averages of triplicates. bar ± SEM. pUC ori: origin of replication of pUC-type; sup t-RNA: suppressor t-RNA; BGH pA: bovine growth hormone polyadenalytion signal; CMV Pr: CMV promotor

3-[bis(3-aminopropyl)amino]propyl-[2-[[2-[di(tetradecyl)amino]-2-oxo-ethyl]amino]-2-oxoethyl] ammonium; hydrochloride (DMAPAP, also called RPR209120) was synthesized in our laboratory as described [24]. YUK1350 (also called RPR206252) was synthesized in our laboratory as described [25]. The chemical structures are presented in Figure 1.

### Plasmids

Plasmid free of antibiotic resistance version 4 (pFAR4) gene vectors are antibiotic-free plasmids that are propagated using a specific *Escherichia coli* (*E. coli*) strain which is auxotrophic for thymidine [20]. The pFAR4-Luc plasmid encodes Luc expressed from the cytomegalovirus (CMV) promoter [20]. The pFAR4-IL-12 plasmid encodes the murine secreted IL-12 cytokine expressed from the CMV promoter. The IL-12 DNA sequence, synthesized by Geneart (Life Technologies SAS, Courtaboeuf, France), encodes the two P40 and P35 IL-12 subunits fused with an elastin linker (VPGVGVPGVG, pUN01-mIL12p40p35, Invivogen, Toulouse, France). Both resulting plasmids were entirely sequenced and purified using Endofree Plasmid purification kits (Macherey Nagel).

### Cationic liposome preparation

Cationic liposomes composed of DOPE, DMAPAP, YUK1350, or YUK1390 were obtained by the ethanolic injection method as described [26]. All the lipids were dissolved in ethanol (EtOH) at a concentration of 10 mmol/L and dropped in stirred water. After EtOH evaporation, liposomes were then stored at 4°C for further experiments. The formulation compositions tested are given in Table 1.

**Table 1.** Lipid components for the various liposome formulations

**Formulation**	**DOPE (mol%)**	**DMAPAP (mol%)**	**YUK1390 (mol%)**	**YUK1350 (mol%)**
DOPE:DMAPAP	50	50	0	0
DOPE:DMAPAP:YUK1350	50	25	0	25

### Lipoplex transfection

Typically, lipoplexes were obtained by dropwise addition of an equal volume of diluted pFAR4-Luc or pFAR4-IL-12 (in 10% glucose, 40 mmol/L NaCl) into a diluted solution of cationic liposomes to reach an amine/phosphate ratio = 6. The lipoplexes were left at room temperature for 30 min before being incubated with CT26 cells in DMEM + FBS for 24 h at 37°C using 1μg of plasmid per well. The transfection efficiency was evaluated by measuring IL-12 in the supernatant [DuoSet enzyme-linked immunosorbent assay (ELISA) mouse IL-12 p70]. Results were normalized according to the protein content quantifying with Pierce™ BCA Protein Assay Kit (Thermo Fisher Scientific). Therefore, the amount of IL-12 was expressed as pg IL-12/μg of proteins.

### HIFU experiment *in vitro*

A piezoelectric ultrasound transducer was used at 1.5 MHz with a spherical curvature of 20 mm and an active diameter of 25 mm (Imasonic, France) leading to a typical focal spot size of 1.2 mm × 1.2 mm × 6.2 mm. The transducer is coupled to the *in vitro* medium or the animals through a balloon filled with degassed water and closed by a latex membrane (Figure S2). Water was degassed prior to each experiment for 15–20 min. The transducer is also connected by a cable to a single-channel radiofrequency amplifier from Image Guided Therapy (France) with a programmable interface allowing to shoot custom sequences to measure in real-time the transmitted and reflected electrical powers (Figure S3).

Different HIFU sequences were tested *in vitro* on CT26 cells to choose the appropriate parameters for further studies. These parameters are detailed in Table 2. Briefly, on the day of experiment 5 × 10^5^ CT26 cells were loaded in a tube which was placed at the focus point of the transducer through a layer of ultrasound coupling gel with a reference tube near it. The temperature was also controlled during the experiment. The cell pellet was suspended in phosphate buffer saline (PBS) to perform an Annexin V viability test.

**Table 2.** HIFU parameters for HIFU treated CT26 cells *in vitro*

**HIFU condition**	**Amplitude (%)**	**Power (MPa)**	**Power (W)**	**Pressure (MPa)**	**Pulse duration (s)**	**Delay (s)**	**Total time (s)**	**Duty cycle (%)**
30T 5 s	30	1.7	5.5	1.7	0.1	0.2	5	33.3
40T 10 s	40	2.1	9.0	2.1	0.1	0.2	10	33.3
60T 10 s	60	3.0	20.0	3.0	0.1	0.2	10	33.3
80T 10 s	80	3.9	32.7	3.9	0.1	0.2	10	33.3

MPa: Megapascal; W: Watt; s: second

### Annexin V binding assay

Cell viability after HIFU treatment was determined using the fluorescein isothyocyanate (FITC)-Annexin V Apoptosis Detection Kit II (BD Pharmingen). Cells were stained with FITC-Annexin V and propidium iodine (PI) following the manufacturer’s instructions. After that, 10,000 events were recorded per sample on Guava^®^ easyCyte™ Flow Cytometer. The CT26 cells were gated in the forward scatter (FSC)-side scatter (SSC) dot plot, then the viable/early apoptotic/dead cells were determined in the green and red channels using the Guavasoft 3.1.1 software (Figure S4).

### Tumor graft

CT26 tumor-bearing mice were used for the evaluation of transfection efficacy while CT26-Luc tumor-bearing mice were used for the anti-tumor effect of the HIFU. The CT26-Luc cell line was cultured in complete DMEM supplied with 0.4 mg/mL of Geneticin (G418 sulfate, Gibco Life Technologies at 37°C) in a 5% CO_2_-humidified atmosphere. A subcutaneous CT26-Luc tumor was resected, placed into sterile phosphate buffer (Dulbecco’s phosphate buffer, Sigma), cut into fragments of 30 mm^3^, and inserted subcutaneously using a 12-gauge trocar (38 mm) into the mouse flank or in the back of the mouse. The tumor growth was monitored with a caliper for 8–11 days before further experiments. The volume was calculated according to the formula:

$$\text{Volume}=\frac{\text{Width}\times \text{Width}\times \text{Length}}{2}$$

### Cancer treatment by immunotherapy *in vivo*

In this approach, pFAR4-IL-12 was injected alone or complexed with lipids intratumorally 24 h before HIFU ablation to transfect viable cells and obtain IL-12 secretion before HIFU application. pFAR4-IL-12 (50 μg in 25 μL 5% glucose) was injected intratumorally. Six hours after injection of the plasmid, 25 μL of liposome DOPE:DMAPAP:YUK1350 (50:25:25 mol%) containing 0.16 μmol of cationic lipid YUK1350 in PBS were injected at the same site day 0 (D0). On the next day, the tumor was treated with HIFU 80T for 60 s [1 day after treatment (D+1)]. At D0 and D+1, mouse sera were collected to determine the IL-12 and IFN-γ production in the circulation. Tumor growth and body weight of the mice were monitored regularly until the end of the experiment or when the tumor reached more than 10 mm in one dimension. At the end point, mice were sacrificed and spleens were collected to analyze spleen lymphocytes activation. To do that, splenocytes were collected and 2 × 10^5^ cells were cocultured with 10^4^ CT26-Luc cells for 48 h.

### ELISA test

Murine IFN-γ, TNF-α, and IL-12 levels were quantified in the supernatant of cell cultures or the mouse sera using the ELISA kit from the R&D system (DuoSet ELISA mouse IL-12 p70, TNF-α, or IFN-γ). The amount of each cytokine was calculated based on a calibration curve prepared at the same time with the samples.

### Optical imaging

Luc level in the tumor was monitored by optical imaging 20 min after injection intraperitoneally (i.p.) of 2 mg luciferin substrate. Bioluminescence was recorded for 10 min with the Optima camera (Biospace Lab). The quantification was performed over a region of interest (ROI) applied to the tumors using M3 vision software and the results were presented as the total bioluminescence signal in the chosen ROI normalized with the background signal in the same area [bioluminescence imaging (BLI)/bg].

### Splenocytes dissociation and proliferation

Extracted mice spleen were washed with sterile PBS and sterile Roswell Park Memorial Institute (RPMI) medium, then cut into small pieces and grinded with a syringe piston rod. The mixture was passed through a 70 μm strainer and the red blood cells were then lysed using lysis buffer (2 mmol/L Tris, 0.84% NH_4_Cl, pH 7.2) for 5 min. Splenocytes were collected after centrifugation at 1,600 rpm for 5 min at 20°C and resuspended in RPMI medium completed with 10% of FBS. At this step, about 2–3 × 10^8^ cells could be collected. The splenocytes were cultured in a 10 mL complete RMPI medium on 100 mm × 15 mm Petri dishes (P5606-400EA, Sigma Aldrich, France) at about 2 × 10^6^ cells/mL with 5% CO_2_ at 37°C.

### Statistical analysis

GraphPad Prism software was used to analyze the data and determine statistical significance between groups. Data are presented as mean ± standard error of the mean (SEM). One-way or two-way ANOVA test was used. Dunnett’s multiple comparisons test was also applied to compare each treatment to the control group. The meaning of the symbols used in the graphs was as follows: ns: not significant, *P* > 0.05; * *P* ≤ 0.05; ** *P* ≤ 0.01; *** *P* ≤ 0.001; **** *P* ≤ 0.0001.

## Results

### *In vitro* preliminary optimization and evaluation of pFAR4-IL-12 delivery and transfection, cationic lipid administration and HIFU

All plasmids used in this study were derivatives of the pFAR4 backbone which is an antibiotic-free gene vector. The pFAR4 plasmids present multiple advantages. They are devoid of dispensable and potentially inflammatory prokaryotic DNA sequences. They have a small size which is favorable for long-term transfection, and they allow the insertion of a multiple cloning site (MCS) for easy insertion of any eukaryotic expression cassette [27]. In this study, two pFAR4 derivatives were used. The pFAR4-Luc encoding the *Luc* gene allows monitoring the assessment of cell transfection efficacy *in vitro* or in mice using bioluminescence [28]. The pFAR4-IL-12 was designed to provide DCs activation and maturation in tumor-bearing mice. IL-12 (referred to as “P70”) is a heterodimeric cytokine composed of the P35 and P40 subunits. For this study, IL-12 was expressed as a P40:P35 fusion protein (Figure 1A).

The cationic lipids used are linear polyamine-based lipids (Figure 1B) which can interact very efficiently with DNA [13]. Cationic liposomes composed of DOPE and DMAPAP (50:50 mol%) were shown to be as efficient as lipofectamine for transfection *in vitro* [13]. As plasmid DNA does not efficiently transfect cells without a carrier, cationic liposomes made with DMAPAP were used to evaluate the validity of IL-12 expression *in vitro* from the pFAR4-IL-12. As the cationic lipid YUK1350 was previously shown to interact with TLR2 [12, 15], we selected it to be used as an adjuvant *in vivo*. To ensure that YUK1350 was able to properly deliver the plasmid into the cells, we compared the transfection efficiency of pFAR4-IL-12 delivered with the formulation DMAPAP/YUK1350 with the transfection mediated by our standard DMAPAP liposomes by quantifying the amount of secreted IL-12 protein. The transfection efficacy expressed as pg of IL-12 per μg intracellular proteins is presented in Figure 1C. The level of IL-12 (< 0.1 pg/mL) was similar for untreated cells or cells incubated with naked pFAR4-IL-12. An amount of 4.9 pg and 7.0 pg IL-12/μg intracellular proteins was measured when adherent or suspended CT26 cells, respectively, were transfected with DOPE/DMAPAP/pFAR4-IL-12 lipoplexes. A similar amount of IL-12 was produced from cells transfected with lipoplexes containing YUK1350 (25 mol%). A minor enhancement of IL-12 production was measured in suspended cells as regard to adherent cells with both DOPE:DMAPAP:pFAR4-IL-12 and DOPE/DMAPAP/YUK1350/pFAR4-IL-12 lipoplexes (8.0 pg/μg and 7.2 pg/μg cell protein, respectively) thanks to a probable enhanced interaction between the cells and the lipoplexes. As there was no statistical difference between the two formulations, we concluded that the incorporation of YUK1350 within the formulation did not alter the transfection efficiency and could be further used for *in vivo* experiments (Figure 1C).

**Figure 2.**
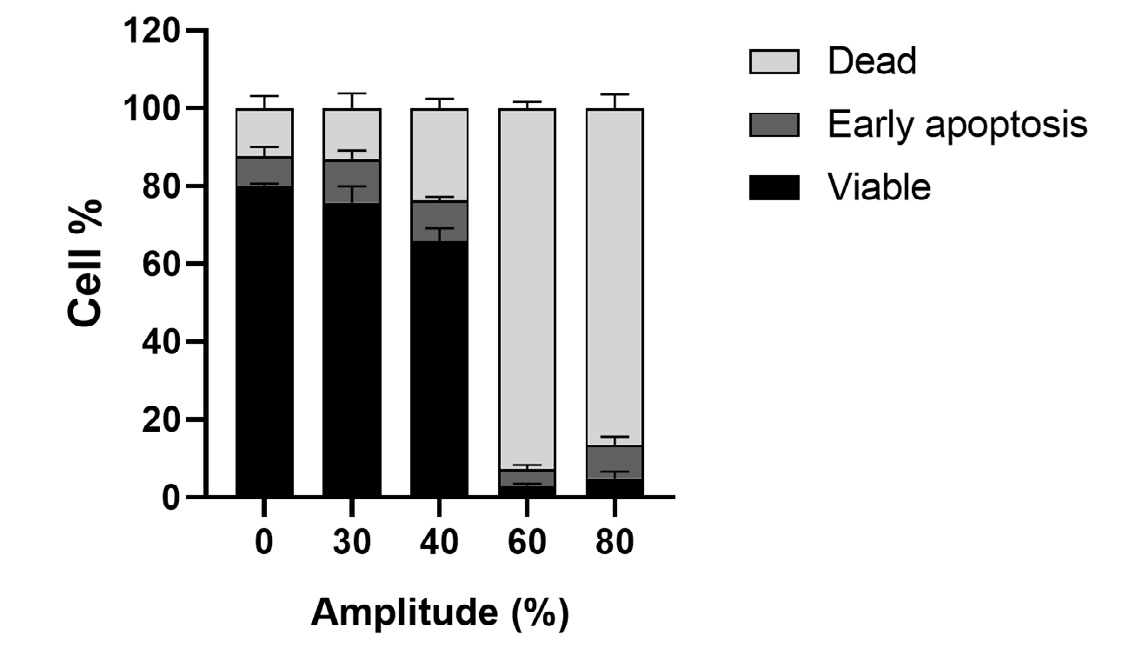
Viability of CT26 cells treated with short-thermal HIFU at various amplitudes for 10 s. 5 × 10^5^ CT26 cells in 50 μL of cell culture medium were incubated at 37°C (0% Amplitude) or treated with short-thermal HIFU at various amplitudes (30% to 80%) for 10 s. Percentage of viable/dead/early apoptosis cells were calculated from Annexin V apoptosis assay 30 min after HIFU treatment. The results are presented as an average of 3 independent experiments (bar ± SEM)

To determine the optimal parameters for HIFU treatment (Figures S2–S3), CT26 cells were suspended in a medium containing either the free plasmid or the lipoplexes. After applying HIFU, the cells were transferred to a plate and cultured for 24 h to determine the proportion of viable, apoptotic, and dead cells. First, an amplitude of 30% of the maximum amplitude of our ultrasound amplifier (called “HIFU 30T”) corresponding to about 1.7 MPa peak negative pressure (PNP) as measured with a calibrated hydrophone in a water tank was applied during 10 s and showed a slight decreased in cell viability to 76%, compared to 80% in the control. By increasing the amplitude to 40% (“HIFU 40T” i.e. about 2.1 MPa PNP) the proportion of viable cells decreased to 66%. The HIFU conditions with 30% and 40% amplitude could be interesting to perform *in vitro* combination experiments on viable cells but would not result in tumor ablation. When HIFU amplitude was further raised to 60% or 80% (3.0 MPa and 3.9 MPa PNP in water, respectively) for 10 s, about 90% of the cells were killed due to hyperthermia. These two last conditions were then considered later for thermal ablation *in vivo* (Figure 2; Figure S4).

### Feasibility and conditions for *in vivo* transfection and tumor ablation

Prior to combining lipoplexes administration and HIFU application *in vivo*, we first assessed the transfection efficacy of our formulations *in vivo*, and we secondly, assessed the HIFU conditions to reach immunogenic death.

**Figure 3.**
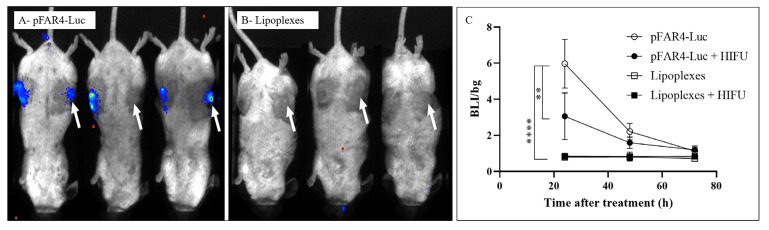
Representative BLI images of mice 24 h after transfection and quantitative representation of intratumor transfection. (A) CT26 tumor bearing-mice were injected intratumorally with 20 μg of free pFAR4-Luc or (B) lipoplexes composed of pFAR4-Luc and DOPE/DMAPAP/YUK1350 treated (right tumors, white arrow) or untreated (left tumors) with “HIFU 80T 30 s” protocol (1.5 MHz, 80% of amplitude, 0.1 s/pulse, and duty cycle of 33.3% for 30 s). For the BLI image, mice were injected by intraperitoneal route with 2 mg of luciferin, and the signal acquisition was performed with an intensified-charge coupled device (iCDD) camera for 10 min. (C) *Luc* gene expression in tumor 1–3 days after transfection with naked pFAR4-Luc or lipoplexes with or without HIFU. Results are presented as BLI signals in the tumor divided by the background. *n* = 3; bar ± SEM; ** *P* ≤ 0.01; **** *P* ≤ 0.0001

For *in vivo* transfection, mice-bearing CT26 tumors were injected intratumorally with either naked pFAR4-Luc (Figure 3A) or DMAPAP:DOPE:YUK1350:pFAR4-IL-12 complexes (Figure 3B) in the presence (right tumor) or absence (left tumor) of HIFU. The HIFU condition 80T for 30 s was chosen as the number of cells in the tumor was much higher than that in the *in vitro* experiment. According to a study from our group, a CT26 tumor fragment of 20–30 mm^3^ was evaluated to contain approximately 9 × 10^5^ tumor cells [29]. If we want to transpose the HIFU parameters *in vivo*, the acoustic absorption of skin and fat tissue leads to a high degree of attenuation and thus lowers the energy delivery, moreover, the center of the tumor should be reached to have an apoptotic effect on all tumor cells. Therefore, we chose the highest amplitude of the HIFU (80%) in a primary experiment. The expression of pFAR4-Luc was evidenced by the bioluminescence signal measured in the tumor 24 h and 72 h after intratumoral injection of free or complexed pFAR4-Luc (Figure 3A and B). In opposite to the *in vitro* transfection results, we found that the complexation of the pFAR4-Luc to cationic lipid led to undetectable transfection. The discrepancy between *in vitro* and *in vivo* results is not surprising and has previously been shown by Nomura et al. [30] using pCMV-Luc/3β-[N-(N’,N’-dimethylaminoethane) carbamoyl] cholesterol (DC-chol) lipoplexes or Coll et al. [31] who injected linear polyethyleneimine (L-PEI)/DNA complexes. Finally, from these results, we concluded that naked pFAR4-Luc was more efficient than lipoplexes for gene transfection *in vivo* in the tested conditions.

The application of HIFU at 80% of maximum amplitude (80T) for 30 s reduced by a factor of 2 the expression of Luc encoded by pFAR4-Luc (Figure 3C) meaning that viable cells were still present in the HIFU-treated tumor. The decreased transfection might be due to the fact that this HIFU condition which induces tumor cell necrosis *in vitro* (Figure 2), was not sufficient to induce complete necrosis *in vivo*. Two other thermal HIFU conditions at 80% of amplitude for 60 s and 120 s (named HIFU 80T 30 s/60 s/120 s) were tested to find the best one which could give a complete ablation of a 150–200 mm^3^ tumor (unshown). From this preliminary study, we could conclude that the thermal HIFU 80T could possibly be applied for tumor necrosis *in vivo*. Moreover, the HIFU 80T for 60 s or 120 s could be chosen for tumor ablation for a tumor volume of about 150 mm^3^.

### Concept of the combined *in vivo* protocol

**Figure 4.**
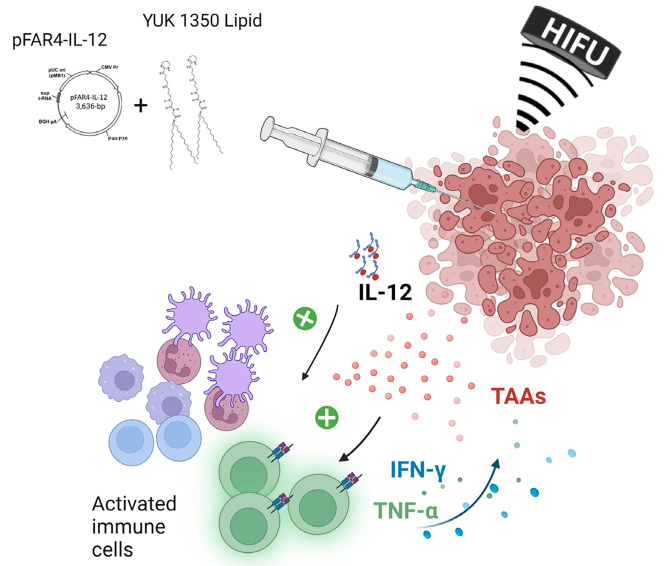
Scheme of *in vivo* combined treatment strategy investigated in this paper. TAAs: tumor associated antigens

The designed combination protocol resulted from the above study. Because DNA formulation by cationic lipids did not enhance transfection *in vivo*, we chose to inject separately lipids and DNA. Indeed, we found that pFAR4-IL-12 was able to induce IL-12 secretion (as shown through *in vitro* experiments) and transgene expression *in vivo* (as proven with the pFAR4-Luc experiments). This thus suggested that pFAR4-IL-12 administered naked *in vivo* should allow an improved production of Th1-derived cytokines, such as IFN-γ, and the activation of cytotoxic effector cells (Figure 4). As the cationic lipid YUK1350 was shown in a previous study to interact with TLR2 receptors, we assumed that its role as an adjuvant could be better obtained via a separate injection. A long application of 80T HIFU for 60 s once or repeated 3 times was chosen to insure in a first combination study thermal ablation of the tumor, resulting in antigen release and APC recruitment (Figure 4).

### Combined *in vivo* protocol

**Figure 5.**
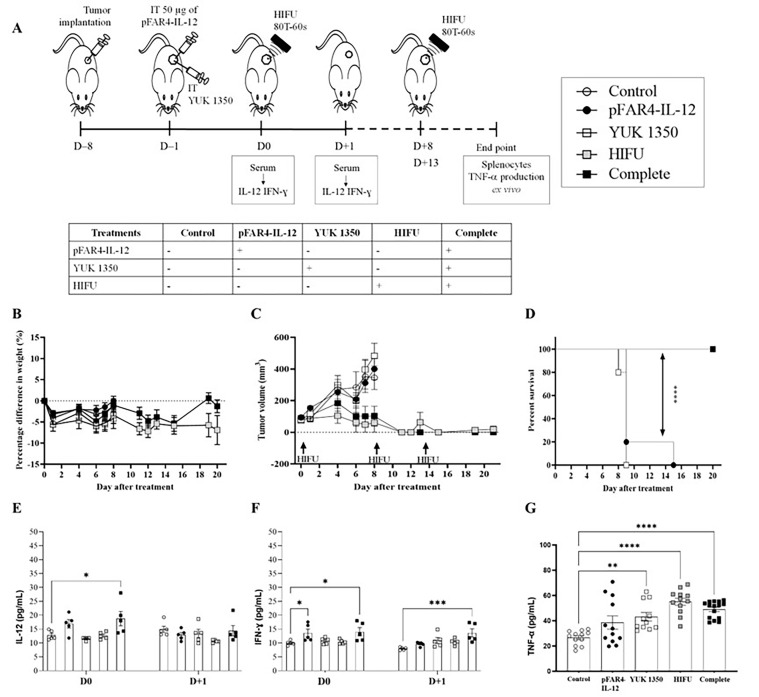
*In vivo* efficacy of the therapeutic strategy. (A) Diagram of the treatment schedule. Balb/C mice were implanted subcutaneously with CT26-Luc tumor fragment on the flank [8 day before treatment (D–8)]. When the tumor reached the appropriate volume, they were injected intratumorally with 50 μg of either naked pFAR4-IL-12 or complexed with liposomes containing 0.16 μmol YUK1350 (D0). On D+1 D+8, and D+13, HIFU 80T 60 s was applied to the tumor (HIFU group or complete group). On D0 and D+1 mouse serum was collected and IL-12 was evaluated by ELISA. Mice were sacrificed when one tumor dimension reached more than 10 mm in one dimension or at the end of the experiment. The different groups studied are listed in the insert. (B) Mice weight as percentage as regard to T0 Mice body weight was monitored regularly and the percentage difference in weight was calculate as function of the weight value of each mouse at D0 [100 × (Value-Baseline)/Baseline]. (C) Tumor volume before and after the treatment. The tumor dimension was measured every 2 days with a caliper and the volume was calculated according to the formula (Width × Width × Length/2). (D) Evaluation of the survival rate of the animals. The Kaplan-Meier method was used for survival analysis. *n* = 4–5; **** *P* ≤ 0.0001. (E, F) Cytokine levels in the mouse sera at D0 and D+1. On D0 or D+1 the mouse serum was collected and cytokine level was evaluated by ELISA. (E) IL-12; (F) IFN-γ; *n* = 5–8; bar ± SEM. (G) TNF-α in the supernatant of splenocytes activated with CT26 after treatment. At the end of each *in vivo* experiment, the splenocytes were collected from sacrificed mice and cocultured with CT26-Luc tumor cells during 48 h. TNF-α secreted in the supernatant was quantified by ELISA. ** *P* ≤ 0.01; *** *P* ≤ 0.001; **** *P* ≤ 0.0001

Naked pFAR4-IL-12 was injected intratumorally at a dose of 50 μg/mouse as published [32]. The cationic liposomes composed of DOPE/DMAPAP/YUK1350 (50/25/25 mol%) were used. Finally, the “HIFU 80T 60 s” protocol was applied for tumor ablation (Figure 5A). The treatment was well tolerated as referred the mouse weight monitored over 8 days (Figure 5B). The weight loss was below 10% in all groups after D0 of treatment and remained stable during the following days. In terms of tumor growth, the tumors in the control group grew from 94 ± 26 mm^3^ on the D0 to 347 ± 168 mm^3^ on D+8. Similarly, in pFAR4-IL-12 and lipid group, the tumors developed at the same rate as the control ones. After 8 days, they reached 411 ± 143 mm^3^ and 449 ± 188 mm^3^ in pFAR4-IL-12 and lipid group, respectively. In contrast, in the HIFU and the complete group, tumor growth was clearly inhibited. Volumes remained similar one week after the last HIFU session (Figure 5C). The effect of HIFU alone repeated three times seems really interesting to provide complete thermal ablation. In terms of survival, a significant difference was obtained between the groups with and without HIFU. Indeed, median survival of 9 *versus* > 20 for the HIFU groups was observed (Figure 5D).

As shown in the schedule diagram (Figure 5A), the level of immunocytokines IL-12 and IFN-γ were quantified 24 h after transfection (Figure 5E, F). We found that the IL-12 level in the serum of mice transfected with pFAR4-IL-12 was 3 times higher than in the control or the lipid group. These results point out that the pFAR4-IL-12 was successfully transfected and IL-12 was secreted into the systemic circulation. However, 48 h after transfection, the level of IL-12 in the serum decreased to the background level in all groups. Moreover, the HIFU conditions leading to a full tumor ablation resulted in the elimination of cells expressing IL-12. Therefore, a lower HIFU condition should be applied to maintain APC viability.

The effect of IL-12, cationic lipid, and HIFU on the production of IFN-γ and TNF-α was also studied. While the level of TNF-α was similar in all groups, indicating either no change or weak modifications under the assay detection limit at this time point, we found that 24 h after the transfection of pFAR4-IL-12, the level of IFN-γ was significantly higher in the serum (6.8 pg/mL and 7.2 pg/mL in pFAR4-IL-12, and complete combined treatment, respectively, *versus* 2.8 pg/mL in the control group). The concentration of IFN-γ did not increase with the cationic lipid or HIFU treatment. Moreover, the level of IFN-γ decreased rapidly to reach the baseline in the pFAR4-IL-12 group 24 h after HIFU application. Interestingly, the IFN-γ concentration remained stable in the complete group 24 h after HIFU. The production of IFN-γ in pFAR4-IL-12 group could be attributed to IL-12-induced enhancement of IFN-γ production by CD4 Th1 and CD8 cytotoxic T lymphocytes as well as NK cells. Meanwhile, 48 h after transfection, the higher IFN-γ level in the complete group as regard to pFAR4-IL-12, lipid, and HIFU group 48 h after transfection suggests that there might be a synergistic effect of these components in the production of IFN-γ.

To determine the impact of local tumor immunomodulation on the systemic immune response, we analyzed the ability of splenocytes to produce IFN-γ and TNF-α *ex vivo*. Splenocytes, taken at the end of the experiment, were co-cultured with CT26 cells to evaluate the specific response. Interestingly, the production of TNF-α from splenocytes in all treated groups was higher than that in the control group. In detail, the concentration of TNF-α secreted from splenocytes in the culture medium for pFAR4-IL-12 and lipid group was about 1.5 times higher than in the control group, and about 2 folds higher for the HIFU and complete treatment groups. The enhancement of TNF-α production suggests that the lymphocytes were activated *in vivo*, meaning that there was a probable immunomodulation effect of IL-12 and the cationic lipid YUK1350 even though, it was not strong enough to elicit an antitumor response. The fact that TNF-α also increased in the YUK1350 group suggests that an inflammatory response has been elicited since monocytes/macrophages are the main suppliers of this master inflammatory cytokine. The application of HIFU alone slightly increased the stimulation of T cells as regard to pFAR4-IL-12 and lipid group (Figure 5G). Even though an additional effect related to the combination of plasmid, lipid, and HIFU was not proven in that experiment, the activation of cytotoxic T cells suggests that the systemic immune response of these treatments would be interesting to evaluate.

### Abscopal effect of the complete treatment

**Figure 6.**
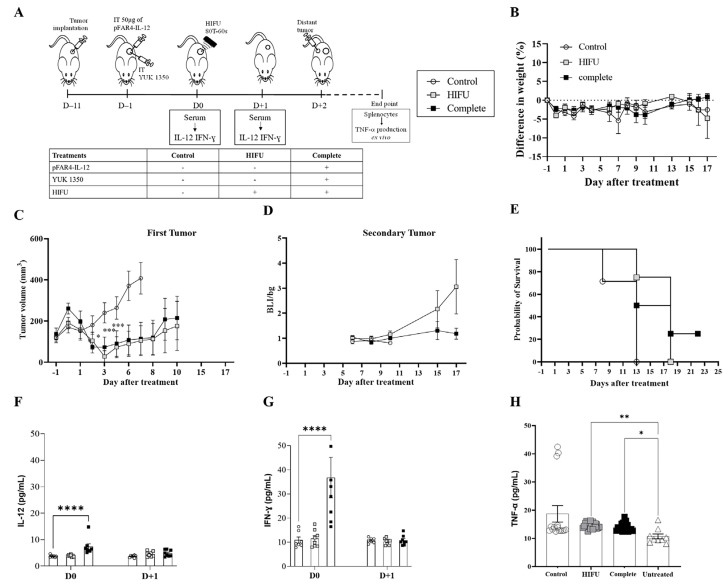
Evaluation of *in vivo* efficacy of the therapeutic strategy as regards to the distal tumor. (A) Diagram of the treatment schedule. Balb/C mice were implanted subcutaneously with CT26-Luc tumor fragment on the flank (D–11). When the tumor reached the appropriate size they were injected intratumorally with 50 μg of pFAR4-IL-12 and/or liposome containing 0.16 μmol YUK1350 (D–1). At D0, HIFU was applied in the tumor (HIFU group or complete group). At D0 and D+1 mouse sera were collected and IL-12 was quantified by ELISA. Mice were sacrificed when one tumor dimension reached more than 10 mm in one dimension or at the end of the experiment. The different groups studied are listed in the insert. (B) Mice weight as a percentage as regard to D–1. Mice body weights were monitored regularly and the percentage difference in weight was calculated as a function of the weight of each mouse at D–1 (100 × (Value-Baseline)/Baseline). (C) Tumor volume of a primary tumor in mm^3^. Tumor sizes were measured 2 days with a caliper and the volume was calculated according to the formula (Width × Width × Length/2) 2-way ANOVA statistical analysis with Dunnett’s multiple comparisons test was applied *** *P* < 0.001, * *P* < 0.05. (D) Bioluminescence ratio of the secondary tumor regarding the background. Secondary tumor was detected by bioluminescence. Mice were injected by i.p. route with 2 mg of luciferin and signal acquisition was performed with an intensified charge-coupled device (iCCD) camera for 10 min. Results are presented as BLI signals in the tumor divided by the background. (E) Evaluation of the survival rate of the animals. The Kaplan-Meier method was used for survival analysis. *n* = 7–8. (F, G) IL-12 IFN-γ; and measured in mouse serum; *n* = 5–8; bar ± SEM. Cytokine level in the mouse serum at D0 and D+1. (H) TNF-α in the supernatant of splenocytes activated with CT26 after treatment. At the end of each *in vivo* experiment, the splenocytes were collected from sacrificed mice and cocultured with CT26-Luc tumor cells for 48 h. TNF-α secreted in the supernatant was quantified by ELISA. ** *P* ≤ 0.01; *** *P* ≤ 0.001; **** *P* ≤ 0.0001

In order to investigate in more detail, the synergistic effect of the HIFU combined with lipid and pFAR4-IL-12, we then chose, to apply HIFU with the same sequence (power and duration) but only once. We had indeed observed in the above study that a single HIFU application was insufficient to prevent tumor recurrence. We, therefore, chose to apply a single HIFU session with or without prior injection of both lipid and pFAR4-IL-12 one day before. We then observed the effect on the growth of the distant tumor implanted 2 days after the beginning of the treatment. The schedule is indicated in Figure 6A, the tolerance of the treatment in Figure 6B.

By reducing the amplitude of HIFU, we could see this time that HIFU was insufficient to remove the tumor and we could evidence an additional effect of the complete treatment. The HIFU significantly limited the primary tumor growth as regards the untreated control (*P* < 0.0001 at D+7) which is an important result for these fast-growing tumors, and the complete group showed similar efficacy. At D+10, CT26 tumors escape from this single HIFU application, which is not the case when injecting plasmid and lipid in addition to this single HIFU application. In this case, at D+17, the difference is still significant (Figure 6C).

To evaluate if an abscopal effect could be obtained thanks to this combined therapy, a secondary tumor was injected (~1–2 mm^3^) at D+2. Even if the bioluminescence signals of the Luc expressing tumors were quite dispersed, we could clearly evidence an absence of tumor growth for the secondary tumor injected for the complete treatment group (Figure 6D), showing a distal effect which was reflected by an improved survival rate for the complete treatment (Figure 6E). A significant increase in TNF-α is observed with HIFU and complete treatment (Figure 6).

## Discussion

IL-12 plays an important role in the activity of the immune system. Transfection of tumor cells with plasmid encoding *IL-12* gene has been shown in many studies to have an antitumor effect. Besides, tumor ablation with HIFU was also found to activate the immune response through the release of tumor antigens and endogenous signals [33]. We, therefore, hypothesized that combining cell stimulation via lipid and plasmid injection followed by antigens release by HIFU ablation could lead to a stronger specific antitumor effect (Figure 4).

We presently show that we can indeed potentialize the effect of HIFU. Some challenges should be addressed though. The local cationic lipid administration should be well defined and adjusted in terms of dosage, the ultrasound parameters to induce tumor cell death while preserving sufficient viable tissue should be optimized, as well as the order of injection of plasmid and cationic lipid formulation, and the application of the acoustically mediated thermal stimulation. The present feasibility study allows to identify several crucial points. First, the expression of the formulated pDNA-gene encoded was too low to induce a measurable IL-12 expression. While the pFAR-Luc allowed to measure Luc transgene expression when the plasmid was injected alone, the expression was not measurable when the plasmid was complexed with the cationic lipid. This was consistent with previously reported studies where immunotherapy with transfection of plasmid encoding IL-12 was not always strong enough to eliminate tumors [8, 34].

As the lipid formulation aimed here to induce a potential TLR2 response, we, therefore, chose to inject it separately. Ultrasound could have potentially been used to favor plasmid release from the lipoplexes, but that would have meant bringing an additional ultrasound sequence within the treatment protocol. In our case, aiming to induce antigen release via cell death, hyperthermia represented the most attractive physical treatment and HIFU is a very good choice as it does burn the tumor mass and microenvironment without impairing healthy tissue thanks to the possibility to focus the beam. The selected HIFU conditions caused tumor necrosis that could be applied for complete tumor ablation. The limitation, though, is the size of the HIFU focal spot. Several focalization spots should be required in the tumor mass to obtain sufficient effect over the whole tumor, and this should ideally be automated with magnetic resonance imaging (MRI) guidance in order to obtain reproducible results and image thermal dose deposition. Such a motorized image-guided protocol would require different equipment and would require a lot more time per animal [35].

Considering the sequence of the different treatments used, Pasquet et al. [36] chose to inject a plasmid encoding IL-12 around the tumor at the same time as electroporation. Such a protocol was difficult to perform with hyperthermia, not knowing whether or not the plasmid would be degraded by heat or another ultrasound-induced effect. We, therefore, chose to inject the plasmid prior to HIFU application in order to stimulate the immune cells prior hyperthermia. This sequence order, together with the replacement of plasmid DNA by an IL-12 mRNA should be further investigated in the future. As our present work was a feasibility study, the complete immune responses have not been investigated. Nevertheless, our results show a potential capacity of pFAR4-IL-12 and its combination with cationic lipid and HIFU in the activation of the immune response. The modification of the cellular tumor environment, such as DCs, lymphocytes infiltration, and cytokine production should be assessed in further studies to better understand the immunomodulation effect of each type of treatment. A preliminary tumor challenge test showed an enhanced survival rate for the complete treatment as regard to HIFU alone. This strongly suggests that the present combination strategy is feasible and should be pursued with some improvements.

## Abbreviations

APC: antigen presenting cell
BLI: bioluminescence imaging
CMV: cytomegalovirus
DCs: dendritic cells
DMEM: Dulbecco’s modified Eagle’s medium
DOPE: 1,2-dioleoyl-sn-glycero-3-phosphoethanolamine
ELISA: enzyme-linked immunosorbent assay
FBS: fetal bovine serum
HIFU: high-intensity focused ultrasound
IFN-γ: interferon-gamma
IL-12: interleukin-12
Luc: luciferase
PBS: phosphate buffer saline
pFAR4: plasmid free of antibiotic resistance version 4
pIL-12: interleukin-12 plasmid
PNP: peak negative pressure
Th1: T-helper 1
TLR2: Toll-like receptor 2
TNF-α: tumor necrosis factor-alpha

## Supplementary materials

The supplementary material for this article is available at: https://www.explorationpub.com/uploads/Article/file/100290_sup_1.pdf.

## Declarations

### Copyright

© The Author(s) 2022.
