# Supplementary material for: Combination of thermal ablation by focused ultrasound, pFAR4-IL-12 transfection and lipidic adjuvant provide a distal immune response
Source: Explor Target Antitumor Ther. 2022 Jun 29;3(6):398–413. doi: 10.37349/etat.2022.00090 (PMC9400762; doi:10.37349/etat.2022.00090)
Supplement: Supplementary file 1 [file etat-03-100290-s001.pdf]

Lipids described in the manuscript

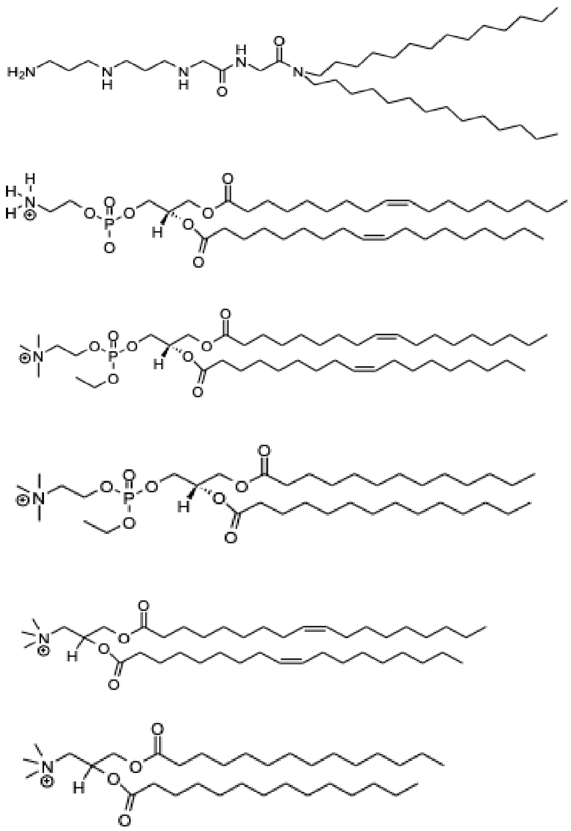

Figure S1. Chemical structures of the lipids described in the manuscript, from the top to the bottom YUK1390, DOPE, DOEPC, DMEPC, DOTAP, and DMTAP

HIFU system

In this study, the frequency of ultrasound was 1.5 MHz given by a transducer with a single spherical curvature of 20 mm and a diameter of 25 mm (Imasonic, France) (**Figure S2A**). Due to the spherical structure, the transducer concentrates the ultrasound beam into a small focal point (**Figure S2B**). The focal point is 1.2 x 1.2 x 6.2 mm at 0.8 cm from the edge of the transducer. Thanks to its latex membrane, a water box allows the coupling of HIFUs. It is connected to a degassing system to eliminate bubbles in the water. The transducer is also connected by a cable to a signal generator and single-channel radiofrequency amplifier from Image Guided Therapy (France) with an interface with touch screen allowing to specify the firing parameters of the sequence and to measure in real time the transmitted and reflected electrical powers. Before experiments, the degassing system was run for 15-20 minutes.

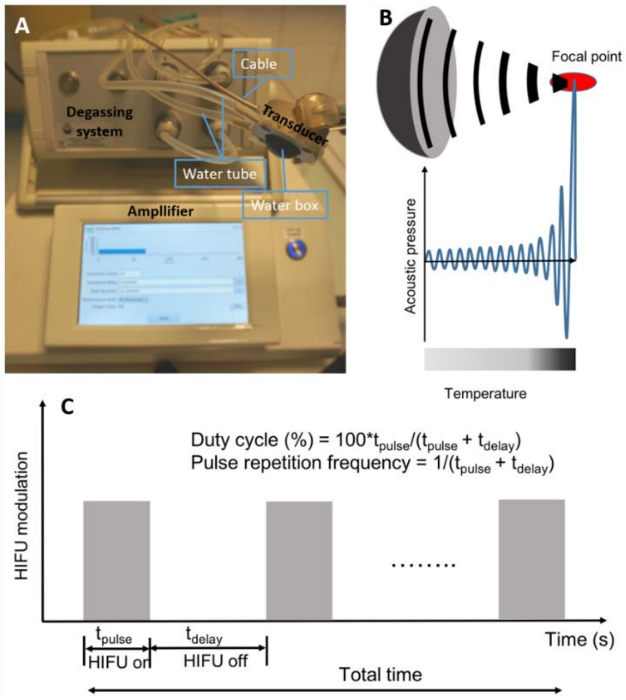

Figure S2. Concept of HIFU. (A) Schematic of a HIFU system. A transducer with a water box is connected to a degassing system to eliminate bubbles in the water. The transducer is also connected to a signal generator and single-channel radiofrequency amplifier. (B) Basic concept of HIFU-induced tissue change by hyperthermia. The transducer with a single spherical curvature concentrates the ultrasound beam into a small focal point. The acoustic pressure as well as the temperature are maximum at the focused point. (C) Time sequence diagram of HIFU modulation and related parameters

As the acoustic frequency is fixed at 1.5 MHz for this transducer, the variable factor, amplitude determines the power created by the transducer to the tissue. While all of the parameters (amplitude, pulse duration, numbers of pulse) define the total energy delivered, the pulse duration and the delay between two pulses take the main role in determining the heat accumulation at the treated tissue (Figure C). The duty cycle is defined as the percentage of the HIFU is turned on in one cycle. Therefore, the higher duty cycle leads to stronger temperature raise at the focused point.

HIFU treatment of CT26 cells in vitro

On the day of experiment CT26 cells were collected with Trypsin - ethylenediaminetetraacetic acid (EDTA) 0.05%, washed twice with sterile PBS and redispered in PBS at concentration of 10^7 cells/mL. 50 µL of cell suspension (5.10^5 cells) was loaded in each 500 µL-PCR tube. HIFU treatment was set as presented in **Figure S3**. In which, an IR lamp was used to control the environment temperature of 37 °C while a forward-looking infrared (FLIR) thermal camera (FLIR 95, FLIR systems, U.S.) was employed for recording real time temperature of the sample. The tube containing cells was placed at the focus point of the transducer through a layer of ultrasound coupling gel.

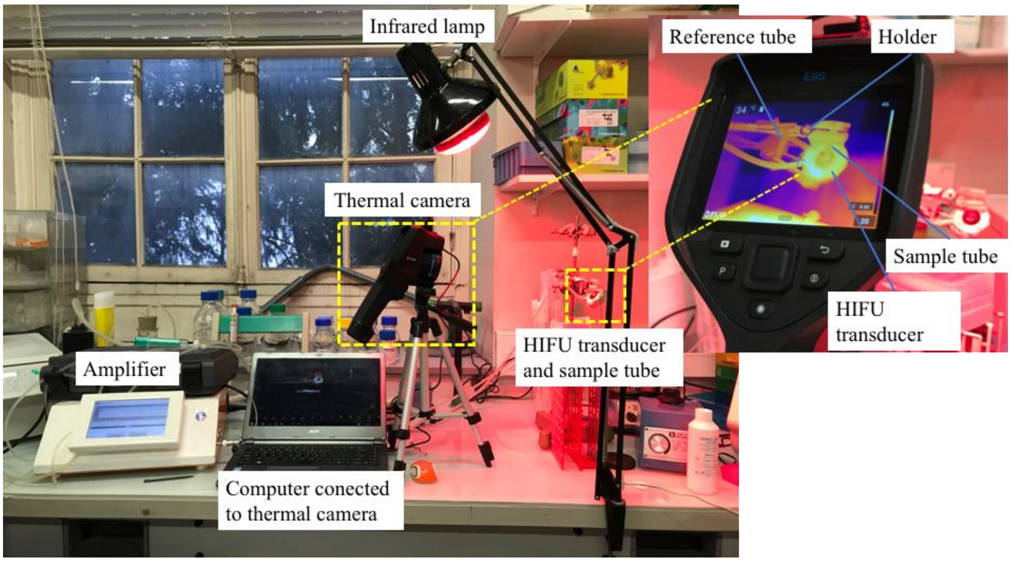

Figure S3. Set up of HIFU treatment *in vitro*.

A HIFU system was set up as presented in Figure A. The tube containing sample was placed at the focused point of the HIFU while a reference tube was place near it. The environment temperature was controlled at about 37 ^{o}C during the experiment with an IR lamp. The real time temperature of the sample was recorded using a thermal camera connected to a laptop.

Annexin V binding assay

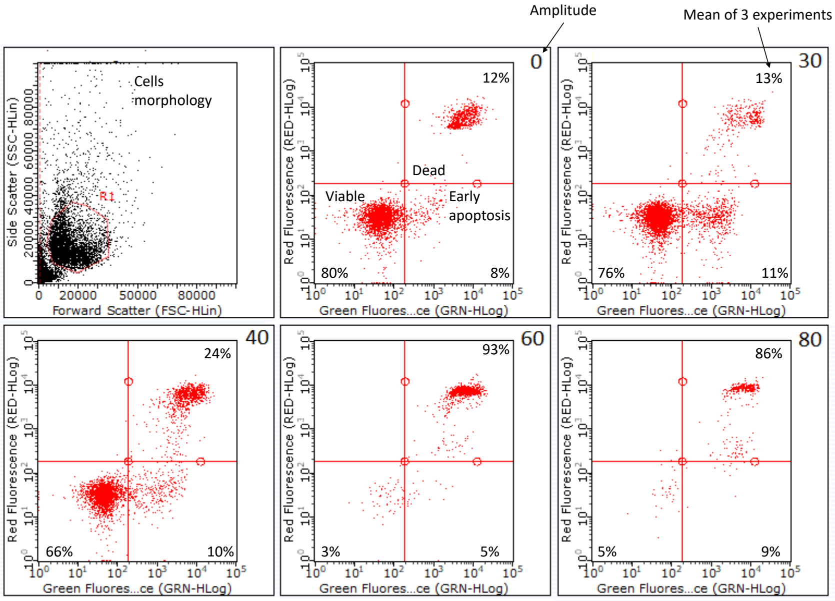

Figure S4. Viability of CT26 cells treated with short-thermal HIFU at various amplitudes for 10 s. 5.10^{5} CT26 cells in 50 µL of cell culture medium were incubated at 37 °C (0% Amplitude) or treated with short-thermal HIFU at various amplitudes (30 to 80%) during 10s. Percentage of viable/ dead/ early apoptosis cells were calculated from Annexin V apoptosis assay 30 minutes after HIFU treatment. Cell morphology (SSC /FSC dot plot) and a representative cytoplot of the red fluorescence (PI) as function of the green fluorescence (Annexin V) was presented for each amplitude condition (0,30,40,60 & 80).
